# Magnitude of short birth interval and associated factors among reproductive age women at rural Kebeles of Gidan Woreda, North Wollo, Amhara, Ethiopia: A cross sectional study, 2023

**DOI:** 10.1016/j.heliyon.2025.e42151

**Published:** 2025-01-23

**Authors:** Bogale Molla, Gizachew Yilak, Adem yesuf, Amira Abdallah, Jemal Derbew, Sewnet Azezew, Getu Engida

**Affiliations:** a School of Nursing, College of Health Sciences, Woldia University, Woldia, Ethiopia; b Department of Midwifery, College of Health Sciences, Woldia University, Woldia, Ethiopia; c Department of Midwifery, College of Health Sciences, Worabe University, Worabe, Ethiopia; d Bati Hospital, Bati, Ethiopia; e Department of Nursing and Midwifery, College of Health Sciences, Debre Berhan University, Debre Berhan, Ethiopia

**Keywords:** Short birth interval, Rural kebeles, Mother, Ethiopia

## Abstract

**Introduction:**

Short birth intervals are associated with negative health outcomes for neonates, infants, and mothers. However, prior studies have not explored the extent of this issue and its associated factors, including family composition, antenatal care visits, and menstrual resumption. This study aimed to assess the magnitude of short birth intervals and identify contributing factors among reproductive-age women in the region.

**Methods:**

A community-based cross-sectional study was conducted among 610 reproductive-age women from April 15 to May 10, 2023. Participants were selected through multi-stage sampling and interviewed using a structured questionnaire. Senior experts evaluated the internal validity of the tool, and a pretest was conducted with 5 % of the participants. The reliability of the questionnaire was assessed using Cronbach's alpha, which was found to be 0.73, indicating acceptable internal consistency. The study analyzed associations between outcome and predictor variables by calculating odds ratios and 95 % confidence intervals. A p-value of <0.05 was considered a cutoff point to assess the significance of associations in the multivariable analysis.

**Result:**

The study found that the magnitude of short birth intervals was 56.5 %. Key factors associated with this outcome included the absence of antenatal care follow-up (AOR = 2.92, 95 % CI: 1.58–5.36), having a female index child (AOR = 1.72, 95 % CI: 1.20–2.48), and poor knowledge about short birth intervals (AOR = 2.52, 95 % CI: 1.74–3.68). Additional predictors included limited postpartum contraception knowledge (AOR = 1.79, 95 % CI: 1.23–2.57), lack of contraception use before the last child (AOR = 1.52, 95 % CI: 1.03–2.24), and having one (AOR = 2.49, 95 % CI: 1.39–4.51) or two female children (AOR = 3.98, 95 % CI: 1.07–7.97).

**Conclusion and recommendation:**

Despite public health interventions tried, short birth intervals remain a major issue in Ethiopia due to factors like limited antenatal care, poor knowledge of birth spacing, and low contraceptive use. To address this, improving access to contraception and raising awareness on family planning and antenatal care are crucial.

## 1 Introduction

A birth interval is the period of time between two successive live births [1]. The 2005 World Health Organization (WHO) technical consultation group on birth spacing recommended a minimum interval of at least 24 months between a live birth and the next conception and a birth-to-birth interval of 33 months or more between consecutive births [2]. WHO defines a short birth interval as an interval between two consecutive live births of less than 33 months [3].

A short birth interval is more common in low- and middle-income nations like those in Africa and Asia, where 17 % of married women of reproductive age have an unmet need for family planning [4]. The result of the Demographic and Health Survey (DHS) of developing countries showed that the deaths of 2 million under-five children that occur each year can be prevented by avoiding birth intervals of under two years [5]. Nigerian DHS data also indicated that 19 % of married women in Nigeria have unmet family planning needs [6].

A short birth interval has adverse perinatal and maternal health consequences. Perinatal consequences include an increased risk of preterm birth, small for gestational age (SGA) [7], infant death [8,9], and low birth weight [10]. Besides increased risks of later pregnancy obesity, increased risk of uterine rupture, gestational diabetes [11,12], malnutrition, anemia, cervical insufficiency, antepartum hemorrhage (APH), and premature rupture of membrane (PROM) [13,14] are among the maternal consequences.

In addition to health complications, short birth intervals contribute to an increase in the total fertility rate (TFR), which poses challenges to a country's development efforts by limiting women's contribution to economic growth [15]. Although Ethiopia's TFR has slightly decreased from 4.8 in 2000 to 4.2 births per woman in 2019, it is above the replacement level of 2.1 children per woman, indicating the need for much more effort to achieve a significant reduction in population growth [16].

Sustainable Development Goal (SDG) number 3 aims to ensure good health and well-being for all, which includes reducing short birth intervals [17]. n line with this goal, the Federal Ministry of Health (FMOH) and the reproductive health departments at regional levels have implemented multi-sectoral approaches at local and national levels to raise awareness about birth spacing, promote breastfeeding, and create an enabling environment for family planning, all aimed at improving maternal and child health outcomes and reducing fertility rates [18].

Although the above strategies have been implemented, more than half of non-first births occur within 3 years of the first birth, and recent estimates showed that the country still experiences higher rates of maternal, neonatal, and infant mortality of 402/100,000, 29/1000, and 47 per 1000 live births, respectively [19]. Hence, a short birth interval directly affects the mother and child's health; it is important to respond to this and to introduce a sufficient interval between births.

Several studies have been conducted in Ethiopia to assess the magnitude and factors associated with short birth intervals [[20], [21], [22], [23], [24], [25]]. However, these studies were primarily limited to urban areas and did not focus on women in rural, resource-constrained settings. For instance, studies in Jijiga [26] and Jimma Zone [18] were limited to city dwellers, making it difficult to generalize the findings to rural populations, as there are notable differences in factors such as education level, awareness of short birth intervals, access to healthcare facilities, and income. Although studies in Gedeb Hasas [24] and Ilubabur [25] districts looked at both urban and rural residents, the results may underestimate the magnitude of the short birth interval due to the difference in socioeconomic variables between urban and rural areas and the difference in cultural background between the study areas. These studies also did not consider the influence of family composition on short birth intervals, despite evidence from research in Bangladesh linking family composition to shorter birth intervals [27].

Additionally, factors such as the number of antenatal care (ANC) visits, resumption of menstruation after the birth of the index child (a limitation highlighted in the study conducted in Tselemti [28]), and participants' knowledge about postpartum contraception, each of which can significantly affect the short birth interval, were not addressed. These gaps further highlight the need for more comprehensive research that includes these factors in rural settings. Furthermore, no studies have yet assessed the magnitude of short birth intervals in the study area, and findings from other regions of the country are inconsistent—short birth interval rates range from 23.3 % in Tselemti to 59.9 % in Jimma Zone. Additionally, while mothers in the lowest wealth quantile are more likely to experience short birth intervals in Dembecha and Arba Minch, the opposite trend is observed in Illubabur. In rural areas cultural norms, socioeconomic status, access to healthcare services and family structures are specifically influencing birth intervals [29].

These variations underscore the importance of localized data on short birth intervals and their contributing factors. Therefore, this study aims to assess the magnitude of short birth intervals and identify associated factors, incorporating previously unstudied variables, among reproductive-age women in Gidan Woreda, Amhara, Ethiopia. This area was chosen due to its limited resources and lack of access to health facilities, which may better represent the magnitude of short birth intervals in rural settings.

## 2 Methods

### 2.1 Study design, area and period

A community-based cross-sectional study was conducted in selected rural Kebeles of Gidan Woreda from April 15 to May 10, 2023. Gidan Woreda is located in the North Wollo Zone of the Amhara Regional State, approximately 630 km from Addis Ababa and 340 km from Bahir Dar. The Woreda consists of 23 Kebeles (22 rural and 1 urban), 5 health centers, and 23 health posts. According to the population projection for Ethiopia from 2014 to 2017, Gidan Woreda had an estimated total population of 179,931, with 91,413 females, 32,388 of whom are in the reproductive age group. The Woreda has a rural population of 168,509 and 41,845 households [30].

### 2.2 Populations

The source population were all reproductive-age women living in rural Kebeles of Gidan Woreda, while the study population included reproductive-age women from selected rural Kebeles who gave live birth within the last 5 years, had at least two consecutive live births, and were available during the data collection period and met the inclusion criteria. Women who had history of stillbirths, twins, or abortions in between the last two successive live births, those who have been living in the study district for less than six months, and those who didn't remember the exact birth date or didn't have a birth certificate or immunization card for their child were excluded from the study.

### 2.3 Sample size determination and sampling technique

**Fig. 1:**
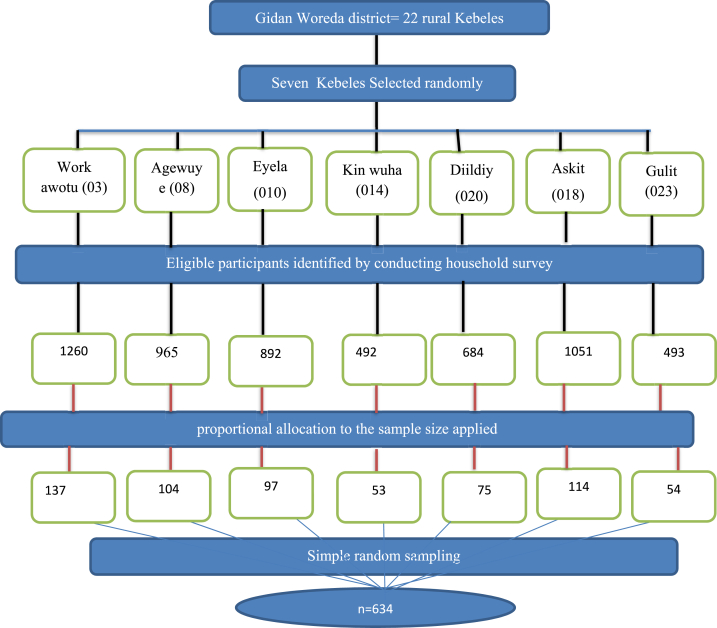
Schematic representation of sampling procedure for study participants on short birth interval and its associated factors among reproductive-age women at rural Kebeles of Gidan Woreda, Amhara, Ethiopia, 2023.

The sample size for the study was calculated using a single population proportion formula, which provide the maximum required sample size to be 634 participants. This calculation was based on a 51.2 % proportion of women with short birth intervals, as found in a previous study in Illubabor [25] with a 95 % confidence interval, a 5 % level of significance, a 10 % expected non-response rate, and a design effect of 1.5 due to multi-stage sampling. The study employed a multi-stage sampling method, starting with the random selection of 7 out of 22 Kebeles in Gidan Woreda using a lottery method, which is 32 % of the rural kebeles and can be representative. A house-to-house visit was conducted to identify eligible women who met the inclusion criteria—those who had given birth to at least two live children within the last five years and were permanently residing in the selected Kebeles. A sampling frame was created for each selected Kebele, and proportional allocation was used to determine the number of participants from each area. Finally, simple random sampling was used to select study participants from the sampling frame. In cases where participants were unavailable during data collection, multiple visits were made to ensure inclusion, and if more than one eligible participant was present in a household, a lottery method was used to select one participant (See Fig. 1).

### 2.4 Data collection tools and procedures

The questionnaire used in the study was developed through a review of various published literatures [20,25,26,31,32], adapting it to the local context to ensure its relevance and applicability. It consisted of five main sections, each focusing on key explanatory variables. Section 1 covered socio-demographic information such as maternal age, marital status, religion, family composition, occupation, education level, access to health facilities, and the husband's occupation and educational status. Section 2 focused on the wealth index. Section 3 dealt with reproductive history, including factors like age at marriage, contraceptive use after the index child, breastfeeding duration, husband's involvement in birth interval decisions, pregnancy planning, place of delivery, child survival, parity, and maternal health services (ANC and PNC) during the index pregnancy. Section 4 assessed women's knowledge of short birth intervals and their perceived disadvantages, while Section 5 focused on participants' knowledge of postpartum contraception.

The data collection process involved face-to-face interviews with eligible women using a standardized, closed-ended questionnaire. To ensure the reliability and quality of data, seven Health Extension Workers (HEWs) and two supervisors (both BSc midwives) were trained for two days by the investigators. Data collectors were instructed to ensure questionnaire completeness and consistency, while supervisors regularly monitored their work. Supervisors checked the accuracy of each filled questionnaire, verifying completion, clarity, and proper respondent identification. To protect participant privacy, names were not recorded on the questionnaires, ensuring anonymity.

### 2.5 Study variables

In this study, the dependent variable was a short birth interval, which was the main outcome of interest. The independent variables included a range of socio-demographic, reproductive, and knowledge-related factors believed to influence short birth intervals.

### 2.6 Operational definitions

**Short birth interval:** it refers to a period less than 33 months between one birth and the next birth [2].

**Index child:** is defined as the child born immediately before the last child [33].

**Good knowledge about postpartum contraception**: there are seven knowledge-assessing questions, and there is one single best answer for five questions and more than one answer for two of the questions; one point was awarded for each correct answer. Correct responses were summed up to get total knowledge scores for each participant, and those who had score ≥50 % were considered to have good knowledge [34].

**Good knowledge about postpartum contraception**: there are seven knowledge-assessing questions, and there is one single best answer for five questions and more than one answer for two of the questions; one point was awarded for each correct answer. Correct responses were summed up to get total knowledge scores for each participant, and those who had score ≥50 % were considered to have good knowledge [35].

**Survival status of index child:** the presence of the index child in life from his/her birth day to the conception of the last child [36].

**Family composition:** The number of sons and daughters a woman had before the birth of her last child [27].

### 2.7 Data quality control

Data quality was ensured through several careful measures. A pretested questionnaire was used, which was carefully designed to capture relevant information. To maintain reliability, the original questionnaire in English was translated into Amharic (the local language) and then back-translated into English by language experts, ensuring consistency between both versions of the tool. The internal consistency of the questionnaire was assessed using Cronbach's alpha, which yielded a value of 0.73, indicating an acceptable level of reliability.

Before data collection began, all data collectors and supervisors underwent two days of training. This training focused on the study's objectives, the data collection procedures, proper handling of data, and how to approach respondents to ensure they felt comfortable and understood the questions. A pre-test was conducted among 5 % of the population (32 reproductive-age women) from Wuraf Kebele in Gubalafto Woreda, an area with similar socio-demographic characteristics. Based on the feedback from the pre-test, necessary modifications were made to the questionnaire.

Senior experts, who reviewed the clarity, relevance, and formulation of the questions to minimize bias and ensure that the tool accurately measured the intended concepts, evaluated the internal validity of the data collection tools. During the data collection process, data collectors introduced the study, provided clarifications to participants as needed, and made sure all questions were properly understood.

Supervisors and the investigator conducted day-to-day on-site supervision, monitoring the activities of each data collector to ensure the quality of data collected. Additionally, the collected data was reviewed daily for consistency and completeness, ensuring that no errors or missing information compromised the study's findings.

### 2.8 Data processing and analysis

The collected data was carefully checked for completeness, coded, and then entered into EpiData version 4.6 before being exported to SPSS version 26 for analysis. Descriptive statistics, including frequencies, percentages, mean, median, and standard deviation, were calculated, and the results were presented through text, tables, and figures. To assess the adequacy of the model, observed and expected frequencies were checked, with no zero cells observed and expected frequencies greater than 5 %. The goodness of fit was tested using the Hosmer-Lemeshow statistic, which resulted in a value of 0.58, indicating an acceptable fit.

To check for multicollinearity among independent variables, both the variance inflation factor (VIF) and tolerance tests were conducted. The maximum VIF was found to be 4.13, and the maximum tolerance value was 0.84, suggesting that there was no significant multicollinearity among the predictors.

For assessing the strength of the association between the independent variables and the outcome (short birth interval), the odds ratio with a 95 % confidence interval (CI) was computed. Variables with a p-value of less than 0.2 in the bivariable binomial logistic analysis were included in the multivariable binomial logistic regression to adjust for potential confounders. Finally, a p-value ≤0.05 with a 95 % confidence interval for the adjusted odds ratio was used to determine statistical significance in the multivariable analysis.

## 3 Results

### 3.1 Socio-demographic characteristics of the study participants

A total of 610 eligible women participated in the study, achieving a 100 % response rate**.** The average age of the participants was 34.53 years (with a standard deviation of ±6.2). Most participants were followers of the Orthodox religion 604 (99 %), while the remainder were Muslims. In terms of education, 244 (40 %) had received primary education**.** Over half of the participants (59.3 %) identified as housewives**,** and nearly 42.3 % belonged to the lowest wealth quintile. Interestingly, a quarter of the respondents reported having married before reaching the legal age of marriage (Table 1 provides further details.).

**Table 1:** Socio-demographic characteristics of respondents among reproductive-age women at Gidan Woreda, Amhara, Ethiopia, 2023 (n = 610).

S.no	Variable	Categories	Frequency	Percentage
1	Age of respondent	20–24	27	4.4
		25–29	114	18.7
		30–34	186	30.5
		35–39	126	20.7
		40–49	157	25.7
2	Marital status of respondent	Married	576	94.4
		Divorced	20	3.3
		Widowed	14	2.3
3	Educational status of respondent	Unable to read and write	88	14.4
		Able to read and write	157	25.7
		Primary education	244	40.0
		Secondary education	100	16.4
		College education and above	21	3.5
4	Occupation of respondent	Employed	21	3.4
		Housewife	362	59.3
		Merchant	37	6.1
		Farmer	190	312
5	Age of respondent during marriage	12–18	154	25.3
		19–24	398	65.2
		≥25	58	9.5
6	Husband educational status	Unable to read and write	44	7.2
		Able to read and write	238	39.0
		Primary education	282	46.2
		Secondary education	25	4.2
		College education and above	21	3.4
7	Husband's occupation	Employed	30	4.92
		Farmer	437	71.64
		Merchant	114	18.69
		Daily laborer	29	4.75
8	Time needed to visit health facility	≤30	234	38.4
		31–60	196	32.1
		≥61	180	29.5

#### 3.1.1 Household socio economic status of respondents (wealth index)

Of the total respondents, nearly half (42.3 %) were in the lowest wealth quintile**,** 29.2 % were in the middle wealth quintile, and the remaining participants were in the highest wealth quintile**.**

### 3.2 Reproductive history of respondents

More than half of the respondents **(**60.7 %**)** had used modern contraception before conceiving their last child. Additionally, over half of the participants (56.1 %**)** had given birth to fewer than four children. However, a significant portion of the study population lacked essential maternal healthcare: 24.9 % did not receive antenatal care (ANC)**,** and nearly half **(**46.1 %**)** had no postnatal care (PNC) follow-up. Thirty one point six percent **(**31.6 %) of respondents delivered their index child at home, while 60.2 % had planned their last pregnancy. Furthermore, 41.6 % of the women made decisions about their birth interval in consultation with their husbands. (Table 2 provides additional details).

**Table 2:** Reproductive history of respondents among reproductive-age women at Gidan Woreda, Amhara, Ethiopia, 2023 (n = 610).

S.no	Variable	Categories	Frequency	Percentage
1	Age of participant at index delivery	17–24	121	19.8
		25–29	233	38.2
		30–34	161	26.4
		≥35	95	15.6
2	Parity	2–4	342	56.1
		≥4	268	43.9
3	Number of living children	≤2	319	52.3
		>2	291	47.7
4	ANC follow up during index delivery	No	152	24.9
		Yes	458	75.1
5	Number of ANC follow up	≤2	351	57.5
		>2	107	17.5
6	PNC follow up during index delivery	No	281	46.1
		Yes	329	53.9
7	Place of delivery of the index child	Home	193	31.6
		Institution	417	68.4
8	Sex of index child	Male	336	55.1
		Female	274	44.9
9	Survival status of index child	Dead	62	10.2
		Alive	548	89.8
10	Menses resumption after birth of index child	Early	316	51.8
		Late	294	48.2
11	Pregnancy intention	Unintended	243	39.8
		Intended	367	60.2
12	Decision on birth interval	Mother	140	23.0
		Husband	216	35.4
		Both	254	41.6
13	Use contraceptive before conception of the last child	No	240	39.3
		Yes	370	60.7
14	Knowledge towards postpartum contraception	Poor knowledge	292	47.87
		Good knowledge	318	52.13

#### 3.2.1 Breast feeding duration of respondents

**Fig. 2:**
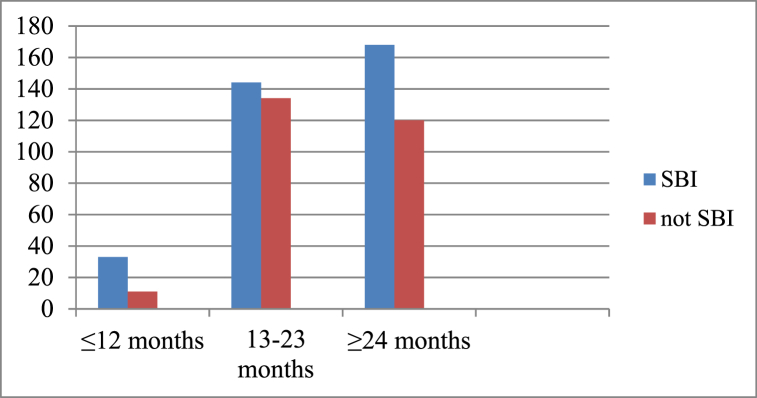
A graph showing relation of breast feeding duration with short birth interval among reproductive age women at rural Kebeles of Gidan Woreda, Amhara, Ethiopia, 2023 (n = 610).

Among the total respondents, **51.1 %** of those who breastfed their child for 13–23 months experienced a short birth interval (Fig. 2).

### 3.3 Magnitude of short birth interval

**Fig. 3:**
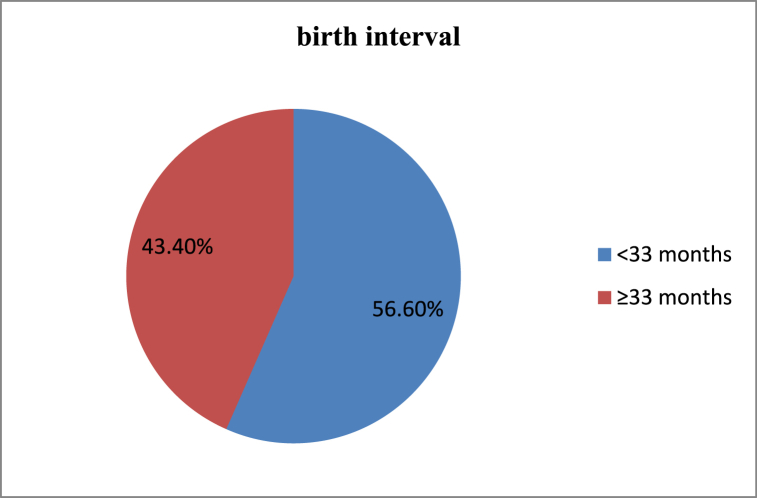
A graph showing duration of birth interval among reproductive age women at rural Kebeles of Gidan Woreda, Amhara, Ethiopia, 2023 (n = 610).

Among the total study participants, the magnitude of the short birth interval was found to be 56.5 % (345 participants), with a 95 % confidence interval of (52.3%–60.9 %). The mean birth interval in the study was 31.5 months, with a standard deviation of ±7.5 months (Fig. 3).

### 3.4 Knowledge of participants towards short birth interval

Almost three-fourths of the respondents **(**74.8 %**)** had heard about short birth interval, but more than half **(**58.9 %**)** had poor knowledge regarding the concept. Around 50.8 % of the participants knew the normal range for birth intervals. Furthermore, over half of the respondents **(**59.7 %**)** understood that a normal birth interval is important for both the mother's and child's health, while the remaining participants believed that either the mother or the baby could benefit from an appropriate birth interval (Table 3).

**Table 3:** Knowledge of respondents towards birth interval among reproductive-age women at Gidan Woreda, Amhara, Ethiopia, 2023 (n = 610).

s.no	Variable	Categories	Frequency	Percentage
1	Heard about short birth interval	No	154	25.2
		Yes	456	74.8
2	Less than how many months to say short birth interval	<33 months	312	51.1
		≥33 months	298	48.9
3	Optimal birth interval has advantage	No	186	30.5
		Yes	391	64.1
4	Normal range of birth interval	below 3 years	51	8.4
		3–5 years	310	50.8
		Above 5 years	155	25.4
		I don't know	94	15.4
5	Beneficiaries of normal birth interval	Mother's health	129	21.1
		Child's health	117	19.2
		Mother's and Child's health	364	59.7
6	Range of birth interval has health dis-advantage	Below 3years	302	49.5
		3–5 years	38	6.2
		Above 5 years	33	5.4
		Below 3 years and above 5 years	117	19.2
		I don't know	120	19.7
7	Disadvantaged by abnormal birth interval	Mother's health	266	43.6
		Child's health	242	39.7
		Mother's and Child's health	102	16.7
8	Overall knowledge towards short birth interval	Poor knowledge	359	58.9
		Good knowledge	251	41.1

### 3.5 Factors associated with short birth interval

Bi-variable and multivariable binomial logistic regression analyses were conducted to identify factors associated with short birth intervals. The bi-variable binary logistic regression analysis revealed several factors associated with short birth intervals. These included the absence of antenatal care (ANC) follow-up during the index pregnancy, attending fewer than two ANC visits**,** having a deceased index child**,** having a female index child**,** poor knowledge about short birth intervals**,** poor knowledge about postpartum contraception**,** having only daughters**,** and lack of contraception use before conception**.**

In the multivariable analysis, six factors remained significantly associated with short birth intervals**:** absence of ANC follow-up**,** having a female index child**,** poor knowledge about short birth intervals**,** poor knowledge about postpartum contraception**,** having only daughters**,** and lack of contraception use before conception at a p-value <0.05.

Specifically, women who did not receive ANC during their index pregnancy were 2.92 times more likely to have a short birth interval compared to those who did {AOR = 2.92, 95 % CI (1.58, 5.36)}. Additionally, women with a female index child were 72 % more likely to experience a short birth interval than those with a male index child {AOR = 1.72, 95 % CI (1.2, 2.48)}. Poor knowledge about short birth intervals or postpartum contraception increased the likelihood of having a short birth interval by 2.52 times {AOR = 2.52, 95 % CI (1.74, 3.68)} and 79 % {AOR = 1.79, 95 % CI (1.23, 2.57)}, respectively. Women who did not use contraception before conceiving their last child were 1.5 times more likely to have a short birth interval{AOR = 1.52, 95 % CI (1.03, 2.24)}. Finally, women who had only daughters before the birth of their last child were significantly more likely to have short birth intervals, with the odds increasing with the number of daughters (Table 4).

**Table 4:** Bivariable and multivariable logistic regression analysis indicating factors associated with short birth interval among respondents in rural Kebeles of Gidan Woreda, Amhara Regional State, 2023 (n = 610).

S.no	Variable	Categories	birth interval		COR (95%CI)	AOR(95%CI)	P-value
			<33 month	≥33 months			
1	ANC during index pregnancy	No	119	33	3.7(2.4,5.6)	2.92 (1.58, 5.36)	0.001∗∗
		Yes	226	232	1	1	1
2	Number of ANC visit (n = 458)	≤2	174	177	1.04(1.01.,2.87)	0.85(0.53,1.39)	0.53
		>2	52	55	1	1	1
3	Sex of index child	Male	164	172	1	1	1
		Female	184	90	1.93 (1.39,2.68)	1.72(1.2, 2.48)	0.003**∗∗**
4	Survival status of index child	Dead	43	19	1.84(1.05,3.24)	1.01(0.51,1.99)	0.96
		Alive	302	246	**1**	1	1
5	Respondents knowledge about Short birth interval	Poor knowledge	263	99	2.57(1.85,3.58)	2.52(1.74, 3.68)	0.000∗∗∗
		Good knowledge	107	141	1	1	1
6	Knowledge about postpartum contraception	Poor knowledge	195	97	2.25(1.6,3.11)	1.79(1.23,2.57)	0.002∗
		Good knowledge	150	168	I	I	1
7	Contraceptive utilization before conception of the last pregnancy	No	163	77	20.19(1.55,3.06)	1.52(1.03,2.24)	0.032∗
		Yes	182	188	1	1	1
8	Family composition	Boy	45	41	1.53(0.82,2.83)	1.44(0.73,2.58)	0.26
		Girl	114	74	2.14(1.25,3.66)	2.49(1.39,4.51)	0.002∗∗
		Boy, girl	44	37	1.65(0.89,3.09)	1.73(0.87,3.48)	0.12
		Boy, boy	37	42	1.22(0.65,2.3)	1.32(0.66,2.63)	0.42
		Girl, girl	72	25	4.01(2.12, 7.59)	3.98(1.99,7.97)	0.000∗∗∗
		>2 children	33	46	1	1	1

***Note:*** 1: reference group, ∗: p-value <0.05, ∗∗: p-value <0.01, ∗∗∗: p-value <0.001.

## 4 Discussion

This study aimed to assess the magnitude of short birth intervals and identify associated factors among reproductive-age women in rural Kebeles of Gidan Woreda, Amhara, Ethiopia. The findings revealed that more than half (56.6 %) of the participants experienced a short birth interval. Factors significantly associated with short birth interval included absence of antenatal care (ANC) follow-up during the index pregnancy, having a female index child**,** poor knowledge about short birth intervals**,** poor knowledge about postpartum contraception**,** having only daughters, and lack of contraception use before conception of the last child.

This study found that 56.6 % of the respondents had a short birth interval, which is higher than the magnitudes reported in previous studies conducted in districts such as Illubabur (51.2 %) [25], Gedeb Hasas (50.4 %) [37], Dembecha (43.4 %) [23], and Tselemti (23.3 %) [28] in Ethiopia. The discrepancy in findings could be attributed to several factors. First, this study focused exclusively on rural residents, a group that may experience higher rates of short birth intervals due to limited access to family planning services and healthcare facilities. Additionally, differences in awareness levels about short birth intervals may explain some of the variation. For instance, 88.9 % of participants in the Tselemti study [28] reported awareness about short birth intervals, which is significantly higher than in the current study, and greater awareness could potentially contribute to reduced rates of short birth intervals. Socio-demographic differences across study areas could also play a role in these discrepancies.

Moreover, when compared to studies abroad, the finding in this study is notably higher than in Pakistan (22.9 %) [38], rural Bangladesh (24.6 %) [27], and Nepal (23 %) [39] where lower rates of short birth intervals were reported. These differences could be due to variations in socio-economic conditions**,** healthcare access, and the definition of short birth intervals, as the cut-off for short birth intervals in Nepal was <24 months, which differs from the criteria used in this study.

Contrary to the lower rates observed in some of these studies, the magnitude of short birth intervals in Chad was much higher (71 %) [40], which may be linked to very low contraceptive coverage in that country (25.2 %) [41] compared to Ethiopia's contraceptive coverage of 41 % [42]. On the other hand, the results of this study align with findings from other research conducted in Ethiopia (Jimma Zone, 59.9 %) [18]**,** Uganda (52.4 %) [32] and rural India (60 %) [31]**,** hich also reported relatively high rates of short birth intervals. The similarity in these findings might be attributed to the focus on rural populations**,** where healthcare access and awareness may be similarly limited, as well as potential similarities in socio-economic conditions between the participants in Jimma and the current study.

The findings of this study indicate that women who did not use modern contraceptives before conceiving their last child were more likely to have a short birth interval compared to those who had used contraceptives. This result is consistent with studies conducted in various locations, including Arba Minch [20], Gonder [43], Debere Berehan [22], Dembecha [23], Dessie [21], Uganda [32], and rural India [31]. This is obvious that Contraceptive use is considered one of the most effective strategies for preventing short birth intervals, which can help avert negative maternal and child health outcomes, such as increased risks of maternal morbidity, preterm births, and low birth weight.

The likelihood of having a short birth interval was 2.9 times higher among mothers who did not attend antenatal care (ANC) follow-up during their index pregnancy. This finding is consistent with the results of a study conducted in Gondar [43]. One possible explanation for this association is that women who do not attend ANC are less likely to receive essential services such as contraceptive counseling and health education. Lack of access to these services may prevent women from learning about the importance of birth spacing and the potential adverse maternal and child health outcomes of short birth intervals.

Moreover, women with a female index child were more likely to have a short birth interval compared to women with a male index child. This finding aligns with studies conducted in Jimma [18], Gedeb [37], Dessie [21], Nigeria [44] could be deeply ingrained social norms that place greater value on male children. In many cultures, including some in Ethiopia and other countries, males are often seen as the "guardians" of the family—they are viewed as protectors, heirs, and providers [45]. As a result, some women may feel pressured to continue having children until they bear a male child, believing that a son is essential for family honor, lineage, and protection.

Women with poor knowledge about short birth intervals were more likely to experience short birth intervals compared to those with better knowledge. This finding is consistent with the results of a study conducted in Dembecha district [23]. The similarity in these findings may be attributed to the fact that women who have a good understanding of what constitutes a short birth interval, its potential consequences, and the strategies for preventing it are more likely to make informed reproductive choices.

Study participants with good knowledge about postpartum contraception were less likely to experience short birth intervals. This finding is consistent with a study conducted in Nepal [39]. The reason behind this may be that increased awareness of postpartum contraception can lead to higher rates of contraceptive acceptance, which can decrease short birth interval**.**

Lastly, women who had only one girl or two girls were more likely to experience a short birth interval. This finding is supported by a study conducted in Bangladesh [27]. This might be due to rooted in gender norms and values perceived by the community [45]. In some cultures, there is a preference for male children, and having only daughters can lead to pressure on women to continue having children until they have a son [46].

### 4.1 Strength and limitation of the study

Despite its typical limitations, this study aimed to address the magnitude of short birth intervals by focusing exclusively on rural residents and incorporating variables that have not been widely studied in Ethiopia, such as family composition, resumption of menstruation, and knowledge about postpartum contraception. To reduce the risk of social desirability bias**,** data collectors were assigned to areas outside their respective Kebeles, which helped ensure that participants felt more comfortable providing honest responses.

This study also had some limitations, like recall bias since birth interval estimation was mainly based on the mother's memory, and the study did not incorporate qualitative exploration of social and cultural influences on birth interval. Although the data collectors were assigned out of their Kebeles, social desirability bias may not be totally prevented.

## 5 Conclusion and recommendation

More than half of the study participants experienced short birth intervals, a figure that is higher than the national prevalence reported by the Ethiopian Mini-Demographic Health Survey (EMDHS) 2019. Key contributing factors identified include the lack of antenatal care, having a female index child, poor knowledge of short birth intervals and postpartum contraception, having only daughters, and not using contraception.

Given these findings, it is recommended that healthcare professionals and stakeholders work to improve access to contraceptive methods and implement awareness programs focused on the importance of birth spacing, family planning, and ANC. These initiatives can help reduce the incidence of short birth intervals and promote better maternal and child health outcomes. Additionally, there is a need for qualitative research to further explore the social, cultural, and behavioral factors influencing short birth intervals, which could provide deeper insights into how to effectively address these challenges in rural communities.

## CRediT authorship contribution statement

**Bogale Molla:** Writing – review & editing, Writing – original draft, Validation, Methodology, Formal analysis, Data curation, Conceptualization. **Gizachew Yilak:** Writing – original draft, Validation, Methodology, Data curation, Conceptualization. **Adem yesuf:** Writing – original draft, Validation, Methodology, Data curation, Conceptualization. **Amira Abdallah:** Writing – original draft, Validation, Methodology, Data curation, Conceptualization. **Jemal Derbew:** Writing – original draft, Validation, Methodology, Data curation, Conceptualization. **Sewnet Azezew:** Writing – original draft, Validation, Methodology, Data curation, Conceptualization. **Getu Engida:** Writing – review & editing, Writing – original draft, Validation, Methodology, Formal analysis, Data curation, Conceptualization.

## Ethical clearance

All methods were performed in accordance with the relevant ethical guidelines and regulations. The Institutional Ethical Review Board (IERB) of Debre Berhan University, Asrat Woldeyes Health Science Campus (AWHSC), granted ethical approval for the study, with the reference number IRB 01/59/2015.

Before data collection, a written permission letter was obtained from the Gidan Woreda Health Office. Oral informed consent was obtained from all study participants after explaining the objectives of the study. To ensure strict confidentiality, participants' names and addresses were omitted from the questionnaires. Additionally, participants were informed of their full rights to skip any questions or withdraw from the study at any time without any consequence. The study adhered to all ethical principles to safeguard the rights and well-being of participants throughout the research process.

## Consent for publication

Not applicable.

## Availability of data and materials

The datasets used or analyzed during the current study are available from the corresponding author upon reasonable request.

## Funding

This research was conducted without any financial support.

## Declaration of competing interest

The authors declare that they have no known competing financial interests or personal relationships that could have appeared to influence the work reported in this paper.

## Abbreviations

ANC: Antenatal Care
AOR: Adjusted Odds Ratio
AWHSC: Asrat Woldeyes Health Science Campus
COR: Crude Odds Ratio
HEWs: Health Extension Workers

PNCPostnatal care.
