# Supplementary material for: Magnitude of short birth interval and associated factors among reproductive age women at rural Kebeles of Gidan Woreda, North Wollo, Amhara, Ethiopia: A cross sectional study, 2023
Source: Heliyon. 2025 Jan 23;11(3):e42151. doi: 10.1016/j.heliyon.2025.e42151 (PMC11947699; doi:10.1016/j.heliyon.2025.e42151)
Supplement: Multimedia component 1 [file mmc1.docx]

Annex- I፡ Information sheet and consent form

**Introduction:** Hello, how are you? My name is ___________. I am working as a data collector in a research conducted by Bogale Molla_,_ Gizachew Yilak, Adem yesuf_,_ Amira Abdellah, Jemal Derbew, Sewnet Azezew and Getu Engida. The research was supported in collaboration with Debere Berhan University Asrat Woldeyes health science campus, nursing department to assess magnitude of short birth interval and associated factors among reproductive-age women who reside at rural Kebeles of Gidan Woreda. You are invited to participate in this study and I kindly request active involvement.

**Study title:** Magnitude of short birth interval and associated factors among reproductive-age women who reside at rural Kebeles of Gidan Woreda, Amhara, Ethiopia, 2023.
**Objective of the study:** To assess magnitude of short birth interval and its associated factors among reproductive-age women who reside at rural Kebeles of Gidan Woreda, Amhara, Ethiopia, 2023.

**Study period:** April 15–May 10, 2023

**Process of study**: as part of this study different questions are prepared to be completed by you. For unclear questions, if you need clarification you can ask any time. Since your participation in this research is depends on your voluntary basis you have the full right to refuse, to participate, and to stop at any time. To complete these questions at least takes 20-25 minutes.

**Advantage and disadvantage**: There is no payment or any special privilege given for your participation in this study but your honest answer to these questions is very important to complete this study that will have an impact on the reduction of neonatal and maternal morbidity and mortality. Also, you are not obliged to participate or give information you don't want. If you are not feeling good at any time, please don't worry to ask to stop the procedure.

**Confidentiality:** The confidentiality of the information you provided to me will be maintained and couldn't be accessed by a third party but it's used for research only and burnt by the end of the survey. If you have any questions regarding this study, you can call me with **+251932361130**.

Could I have your permission to continue? Yes ­_______________

No ­_______________

Signature of the data collector certifying that informed consent has been given verbally by respondent _________________

Name of Kebele______________ Questionnaire code_________

Data collector name ___________Signature ______Date of data collection____________

# Annex II: questionnaire

Questionnaires of English Version
**Part I: questions for assessing Socio-demographic Characteristics**

| **S.N** | **Questions** | **Response and coding** | **Skip** |
| --- | --- | --- | --- |
| 101 | How old are you? | _______ years |  |
| 102 | What is your marital status | 1. Married 2. Single 3. Divorced 4. Widowed |  |
| 103 | What religion do you follow? | - 1. Orthodox   2. Protestant   3. Muslim   4. Catholic   5. Others (specify)____________ |  |
| 104 | What is your educational status | 1. Unable to read and write 2. able to read and write 3. Primary education 4. Secondary education 5. College education and above |  |
| 105 | What is your occupation? | 1. employee (NGO/go.t 2. housewife 3. merchant 4. student 5. farmer 6. others _____ |  |
| 106 | What is education status of your husband? | 1. Unable to read and write 2. able to read and write 3. Primary education 4. Secondary education 5. College education and above |  |
| 107 | What is your husband's occupation? | 1. employed (NGO/go.t) 2. Farmer 3. Merchant 4. Daily laborer 5. others _____ |  |
| 108 | How many sons and daughters did you have before the birth of the last child? | 1. ________sons 2. _________daughters |  |
| 109 | How much time is taken to visit a nearby health facility? | ________minutes |  |

**Part II: Questions for assessing house hold socio economic status (wealth index)**

| **No** | **Question** | **Response and coding** | **Skip** |
| --- | --- | --- | --- |
| 201 | Owner ship of the house | 1.private  2.rental  3.governmental  4.relative  5.other(specify)______ |  |
| 202 | Main construction material used in exterior walls | 1.cement  2.wood with mud  3.other(specify)______ |  |
| 203 | Main construction material used for the Roof | 1.plastic/modern cornice  2.cement/concrete  3.Corrugated iron/metal  4.other(specify) |  |
| 204 | Main construction material used for the floor of your house | 1.sand  2.Cement  5.Ceramic |  |
| 205 | Did you have a separating room for sleep? | 1.yes  2.no |  |
| 206 | Did you have a separate room for animals? | 1.yes  2.no |  |
| 207 | Can you please tell me if any member of your house have any of the following? | Yes No   1. .Electricity 1 2   2.Clock/watch 1 2  3.Radio 1 2  4.Television 1 2  5.Mobile phone 1 2  6. Home phone 1 2  7.refrigerator 1 2  8.chair 1 2  9.Sofa 1 2  10.Table 1 2  11.Bed with cotton, sponge  Mattress 1 2  12.Electric Mitad/stove 1 2 |  |
| 208 | Does the household own any livestock, herds, other farm animals or poultry? | 1.yes  2.no |  |
| 209 | How many of the following animals do You have? | 1. milk cows, oxen or bulls  \|___\|___\|  2. cattle _______  3. chicken \|___\|___\|  4. goats \|___\|___\|  5. sheep \|___\|___\|  6. donkey or mule \|___\|___\|  7. beehives \|___\|___\| |  |
| 210 | Does your household own any agricultural land? | 1.yes  2.no |  |
| 211 | Where do you get water? | 1. into dwelling 2. outside yard 3. elsewhere |  |
| 212 | What’s the main source of drinking water for the member of your house hold? | 1. Pipe water into dwelling 2. Pipe water into yard 3. piped to neighbor 4. Public tap/stand pipe 5. Protected well/spring 6. Unprotected well/spring   7. Other specify_________ |  |
| 213 | What is the main source of water for other purpose in your house hold like for cooking and washing | 1. Pipe water into dwelling 2. Pipe water into yard 3. piped to neighbor 4. Public tap/stand pipe 5. Protected well/spring 6. Unprotected well/spring 7. Other specify_________ |  |
| 214 | If the water is elsewhere, how long does it take, to go there, get water and come back? | __________minutes |  |
| 215 | When do you wash your hands? | 1.before preparing food  2. after latrine use  3. before serving food  4. Other, Specify____ |  |
| 216 | How do you wash your hand? | 1.by using soap  2.by using ash  3. using only water  4 .other specify_____? |  |
| 217 | What kind of toilet facility do members of your household usually use? | 1. Ventilated improved pit latrine 2. Pit latrine with slab 3. Pit latrine without slab 4. No facility/bush/ 5. Other (spedcify)________ |  |
| 218 | Where is this toilet facility located? | 1. In own yard/plot 2. Elsewhere |  |
| 219 | What type of fuel does your household mainly use for cooking?(multiple answer is possible) | Yes No   1. Electricity 1 2   2.Natural gas 1 2  3.Wood 1 2  4.Charcoal 1 2  5.Animal dung 1 2  Other (specify)­­­­­____________ |  |
| 220 | Do you have a separate room which is used as a kitchen? | 1. Yes 2. No |  |

**Part III: reproductive History assessing questions of the respondents about short birth interval in rural Kebeles of Gidan Woreda, northern Ethiopia, 2023**

| **S.N** | **Questions** | **Response and coding** | **Skip** |
| --- | --- | --- | --- |
| 301 | How old were you when you first married? | _______ years |  |
| 302 | How many total live births did you give? | _______ |  |
| 303 | How many living children do you currently have? | _______ |  |
| 304 | How old were you when your first child was born? | _______ years |  |
| 305 | Did you have any ANC follow-up in index pregnancy? | 1. No 2. Yes | If you say 1, skip to Q307 |
| 306 | How many ANC visits did you have for the index pregnancy? | _______ |  |
| 307 | Did you have any PNC follow-up in the index delivery? | 1. No 2. Yes |  |
| 308 | Where did you give birth for the index child? | 1. Home 2. Institution |  |
| 309 | What is the sex of your index child? | 1. Male 2. Female |  |
| 310 | Did your index child is alive until you conceive the last child? | 1. No 2. Yes |  |
| 311 | Did you breast fed your index child? | 1. No 2. Yes | If you say 1, skip to Q313 |
| 312 | For how many months did you breastfeed your index child? | _____________months |  |
| 313 | Does your last child's pregnancy was planned? | 1. No 2. Yes |  |
| 314 | Who decides birth intervals in the family? | 1. Myself 2. My husband 3. Myself and my husband |  |
| 315 | After how many months were your menses resumed after the birth ofindex child? | _____________months |  |

**Part IV: Short birth interval knowledge assessing questions of study participants about short birth interval and associated factors in rural Kebeles of Gidan Woreda, Amhara, Ethiopia, 2023**

| **S.N** | **Questions** | **Response and coding** | **Skip** |
| --- | --- | --- | --- |
| 401 | When was the birth date of your index child? | **___________(** Day/month/year) |  |
| 402 | When was the birth date of your last child? | **___________(** Day/month/year) |  |
| 403 | Have you heard about short birth interval? | 1. Yes 2. No |  |
| 404 | Who is your source of information about birth interval?? | 1. Health care provider  2. Families and friends  3. Social media |  |
| 405 | How many months do you think is short birth interval?? | _____________ months |  |
| 406 | Does optimal birth interval have a health advantages? | 1. Yes 2. No 3. I don’t know |  |
| 407 | Which birth interval has a health advantage? | 1. Below 3yars 2. 3–5 years 3. Above 5 years 4. I don’t know |  |
| 408 | For whom do you think have a health advantages? | 1. Mother’s health 2. child’s health 3. Mother’s and child’s health |  |
| 409 | Which birth interval has a health dis-advantages? | 1. Below 3years 2. 3–5 years 3. Above 5 years 4. Below 3 years and above 5 years 5. I don’t know |  |
| 410 | For whom do you think have a health dis-advantages? | 1. Mother’s health 2. child’s health 3. Mother’s and child’s health |  |
| 411 | Does utilization of contraception prevents short birth interval? | 1. No 2. Yes |  |

**Part 5: Postpartum contraceptive knowledge assessing questions**

| S.No | Questions | Coding and responses | Skip |
| --- | --- | --- | --- |
| 501 | Do you ever heard about contraceptive | 1. Yes 2. No |  |
| 502 | What is your source of information for FP? | 1. TV 2. Radio 3. Health workers 4. Family and friends |  |
| 503 | Can you mention the method that you know? (More than one answer is possible.) | 1. LAM 2. Condom 3. Pills 4. Emergency contraceptive 5. Injection 6. Implant 7. Intra-utérine 8. Permanent methods |  |
| 504 | Did you use contraceptives after the birth of the index child? | 1. No 2. Yes |  |
| 505 | What time is best to start contraceptive use following childbirth? | 1. Immediately following childbirth 2. Within 42 days after delivery 3. After 42 days following delivery 4. I don’t know |  |
| 506 | Can exclusive breast feeding prevent pregnancy? | 1. No 2. Yes |  |
| 507 | Does IUCD have no effect on infant breast feeding? | 1. No 2. Yes |  |
| 508 | Does oral contraceptive pills given for breast feeding mother? | 1. No 2. Yes |  |
| 509 | What are the benefits of contraceptive (more than one answer is possible) | 1. Prevent unwanted pregnancy 2. Make family happy 3. Birth spacing 4. Limit number of children 5. Improve maternal health |  |
| 510 | Who decides about contraceptive method utilization in the family? | 1. Myself  2. My husband  3. Myself and my husband |  |
| 511 | Where do you get access for FP? | 1. Hospitals 2. Health center 3. Health post 4. Private clinics |  |
